# NLRP3 Inflammasome Modulation by Melatonin Supplementation in Chronic Pristane-Induced Lupus Nephritis

**DOI:** 10.3390/ijms20143466

**Published:** 2019-07-15

**Authors:** Francesca Bonomini, Mariane Dos Santos, Francisco Veríssimo Veronese, Rita Rezzani

**Affiliations:** 1 Anatomy and Physiopathology Division, Department of Clinical and Experimental Sciences, University o of Brescia, Viale Europa, 25123 Brescia, Italy; 2 Interdipartimental University Center of Research, Adaption and Regeneration of Tissues and Organs (ARTO), University of Brescia, 25123 Brescia, Italy; 3 Laboratory of Molecular Biology Applied to Nephrology, Experimental Research Center, Hospital de Clínicas de Porto Alegre, 90035-903 Porto Alegre, Brazil

**Keywords:** Lupus nephritis, oxidative stress, inflammation, melatonin

## Abstract

Lupus nephritis (LN) is a kidney inflammatory disease caused by systemic lupus erythematosus (SLE). NLRP3 inflammasome activation is implicated in LN pathogenesis, suggesting its potential targets for LN treatment. Melatonin, an endogenous indoleamine, is considered an important multitasking molecule that has been reported to have anti-inflammatory effects by inhibiting nuclear factor-kappa B (NF-κB)-mediated inflammatory responses in vivo. This molecule has also protective effects against the activation of the inflammasomes and, in particular, the NLRP3 inflammasome. Thus, this work evaluated the effect of melatonin on morphological alteration and NLRP3 inflammasome activation in LN pristane mouse models. To evaluate the melatonin effects in these mice, we studied the renal cytoarchitecture by means of morphological analyses and immunohistochemical expression of specific markers related to oxidative stress, inflammation and inflammasome activation. Our results showed that melatonin attenuates pristane-induced LN through restoring of morphology and attenuation of oxidative stress and inflammation through a pathway that inhibited activation of NLRP3 inflammasome signaling. Our data clearly demonstrate that melatonin has protective activity on lupus nephritis in these mice that is highly associated with its effect on enhancing the Nrf2 antioxidant signaling pathway and decreasing renal NLRP3 inflammasome activation.

## 1. Introduction

Systemic lupus erythematosus (SLE) is an autoimmune disorder with a pathogenesis that involves multiple organ systems with alternating clinical exacerbations and remissions [1]. Among SLE manifestations, lupus nephritis (LN), also known as SLE nephritis, is one of the most serious, affecting more than half of the patients with SLE [2,3]. LN is characterized by impaired renal function with high expression of inflammatory cytokines and glomerulonephritis leading to proteinuria [4].

In vivo models of SLE are useful tools to study numerous aspects of LN. In the last years, it has been shown that the pristane-induced lupus mouse is the most excellent model for pathophysiological studies in acute and chronic kidney disease. Furthermore, it serves for evaluating different mechanisms which include systemic autoimmunity and related kidney damage. The main feature of the pristane-induced lupus mouse model is its similarity to human LN, characterized by glomerulonephritis with loss of endothelial cells, tubular inflammatory damage and increase of biomarkers of kidney disease such as angiopoietin-2, neutrophil gelatinase-associated lipocalin and kidney-injury molecule-1 [4,5,6,7,8,9,10,11].

In LN, the inflammatory pathways contribute to tissue injury and clinical manifestations. Among these, the NOD-like receptor protein 3 (NLRP3) inflammasome is considered a key player [12]. The NLRP3 inflammasome is increased in LN [13,14] and induces a sustained inflammatory status contributing to disease worsening and organ damage [15]. Therefore, the NLRP3 inflammasome could be another potential therapy target of LN [16]. Moreover, recent evidence showed that NLRP3 contributes to glomerular injuries and proteinuria in LN [17]. To date, therapy for LN has been evolving with a substantial number of trials with promising new agents based on the fundamental pathophysiological mechanisms which focus on reactive oxygen species (ROS) production and mitochondria alterations.

Melatonin (N-acetyl-5-methoxytryptamine), is a bioactive indolamine produced endogenously at dark time by the pineal gland. This molecule, in addition to its chronobiotic properties, possesses important antioxidative and anti-inflammatory actions [18,19,20,21,22].

Moreover, the anti-inflammatory properties of melatonin have also been studied in an LN model [23]. It acts in different diseases by the inhibition of the nuclear factor kappa B (NF-kB) pathway [24], the conservation of the mitochondrial homeostasis and its bioenergetic efficacy, and the reduction of ROS production [19]. Recently, melatonin was reported to inhibit NLRP3 inflammasome [25].

Based on these considerations, in this study, we examined whether the dual inhibition of NF-κB and NLRP3 by melatonin is potent to halt the progression of LN in pristane mouse models.

## 2. Results

### 2.1. Effects of Melatonin Treatment on Morphologic and Morphometric Changes

Experimental groups “control mice”, “melatonin vehicle mice” and “melatonin control mice” showed no kidney alterations and were redefined as “control mice” for morphological, histomorphometrical and immunohistochemical analyses. Experimental groups “pristane-LN mice” and “pristane-LN mice with melatonin vehicle” did not show differences in the renal cytoarchitecture and were redefined as “pristane-LN mice” for the same analysis.

**Figure 1:**
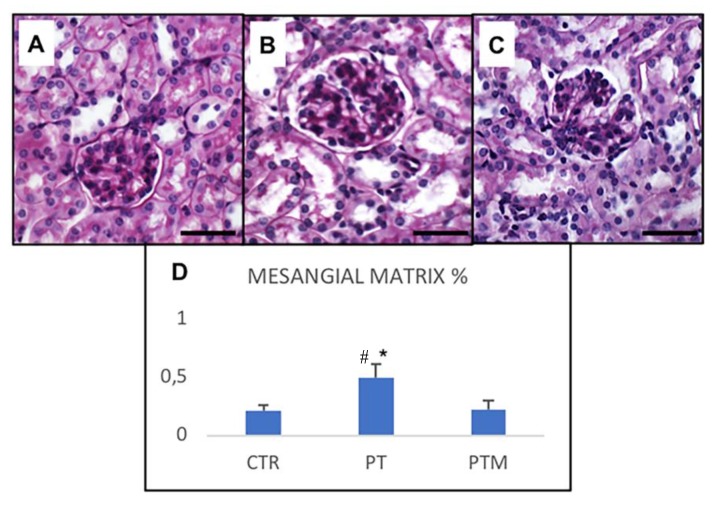
Intraglomerular mesangial matrix and proximal tubules brush border. Photomicrographs showing glomeruli, proximal and distal tubules PAS staining of control mice (**A**), pristane-LN mice (**B**), pristane-LN mice treated with melatonin (**C**). Bar equals 20 μm. The graph (**D**) summarizes the morphometrical analysis of intraglomerular mesangial matrix PAS-positive staining. * # indicate respectively statistically significant differences vs. control and pristane-LN mice treated with melatonin: *p* < 0.05. CTR: control mice PT: pristane-LN mice PTM: pristane-LN mice treated with melatonin.

In pristane-induced LN, glomerulonephritis occurred as shown by PAS staining (Figure 1). In the control group, glomeruli exhibited conventional size and capillary thickness (Figure 1A). Histologic sections of the pristane group showed severe renal damage distinguishable by renal tubules and capillary dilation, with mesangial matrix expansion manifested as mesangial broadening, glomerular atrophy and thickening of capillary walls and basement membrane (Figure 1B). The histopathological changes were reduced in the melatonin-treated group (Figure 1C). Besides glomerular lesions decrease, the intraglomerular mesangial matrix (MMA) was restored. While pristane-induced mice showed a significantly greater amount of MMA, which was assessed as a percentage of the glomerular area (GA), treatment with melatonin decreased the mesangial proliferation, returning the MMA levels to almost those of controls (Figure 1D).

**Figure 2:**
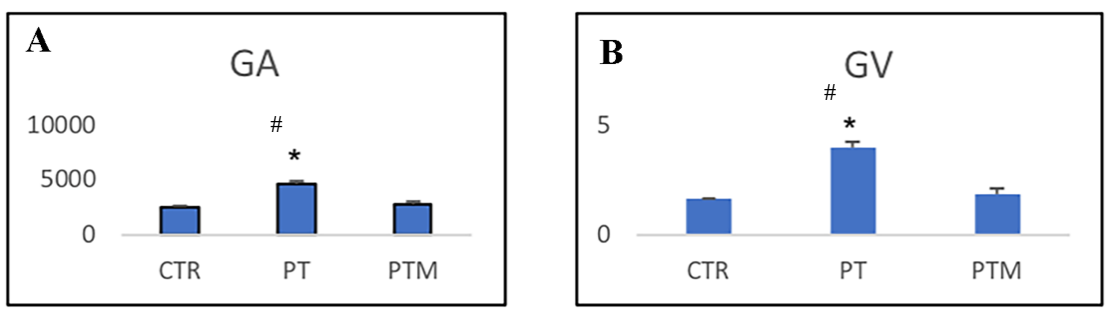
Glomerular morphometric changes in of control mice (CTR), pristane-LN mice (PT), pristane-LN mice treated with melatonin (PTM). (**A**) Glomerular area (GA), expressed as µm^2^, (**B**) Glomerular volume (GV), expressed as 10^6^ µm^3^. * # indicate respectively statistically significant differences vs. control and pristane-LN mice treated with melatonin: *p* < 0.05. CTR: control mice PT: pristane-LN mice PTM: pristane-LN mice treated with melatonin.

The GA and glomerular volume (GV) were significantly larger in pristane-LN mice than in control animals. In melatonin treated animals, the GA and GV were similar to those observed in control mice (Figure 2A,B). Therefore, melatonin prevents the growth of the glomerular capillary tuft and increases in MMA.

### 2.2. Immunohistochemical Analysis of SIRT1 and Nrf2

**Figure 3:**
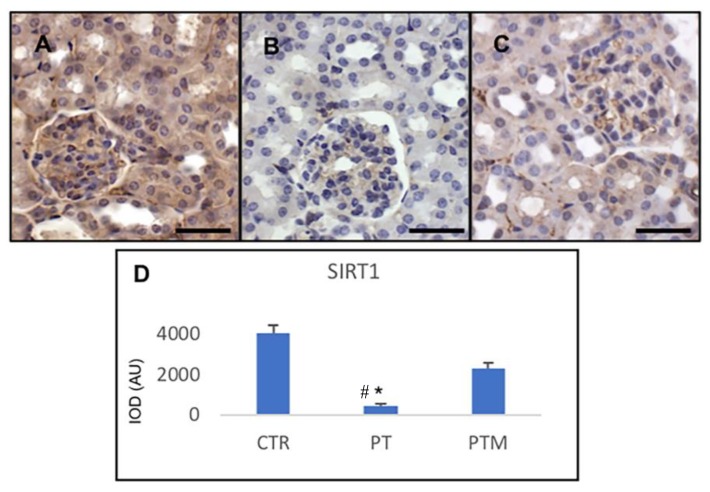
Photomicrographs showing transforming SIRT1 immunohistochemistry of control mice (**A**), pristane-LN mice (**B**) and pristane-LN mice treated with melatonin (**C**). Bar equals 20 µm. The graph (**D**) summarizes SIRT1 immunohistomorphometrical analysis of all experimental groups. * # indicates respectively tatistically significant differences vs. control and pristane-LN mice treated with melatonin: *p* < 0.05. CTR: control mice PT: pristane-LN mice PTM: pristane-LN mice treated with melatonin.

Sirtuin 1 (SIRT1) is a nicotinamide adenine dinucleotide-dependent deacetylase belonging to the class III histone deacetylases and is closely involved in renal physiology; it was abundantly expressed in the kidney of control mice, while in LN mouse model, the expression of this protein decreased significantly. On the other hand, melatonin treatment of pristane-LN mice induced a significant renal increase of the expression of this protective marker (Figure 3).

**Figure 4:**
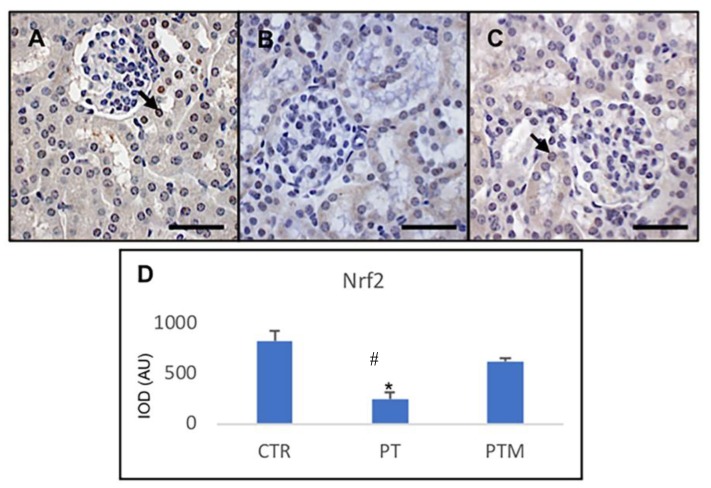
Photomicrographs showing transforming Nrf2 immunohistochemistry of control mice (**A**), pristane-LN mice (**B**) and pristane-LN mice treated with melatonin (**C**). Bar equals 20 μm. The graph (**D**) summarizes Nrf2 immunohistomorphometrical analysis of all experimental groups. Arrows indicates nuclear expression of the protein. * # indicate respectively statistically significant differences vs. control and pristane-LN mice treated with melatonin: *p* < 0.05. CTR: control mice PT: pristane-LN mice PTM: pristane-LN mice treated with melatonin.

Because SIRT1 expression was related to the renal Nrf2 pathway, we evaluated also Nrf2 expression. Nrf2 activation in the tubules of control rats was detected. In particular, immunohistochemistry showed Nrf2 expression in tubular cytoplasm and in the nucleus of distal tubules. Nrf2 positivity was significantly suppressed in the pristane-LN group while the expression of Nrf2 in the melatonin treated group was significantly increased when compared with the LN group in distal tubule nuclei (Figure 4).

### 2.3. Immunohistochemical Evaluation of Inflammatory and Oxidative Stress Status

**Figure 5:**
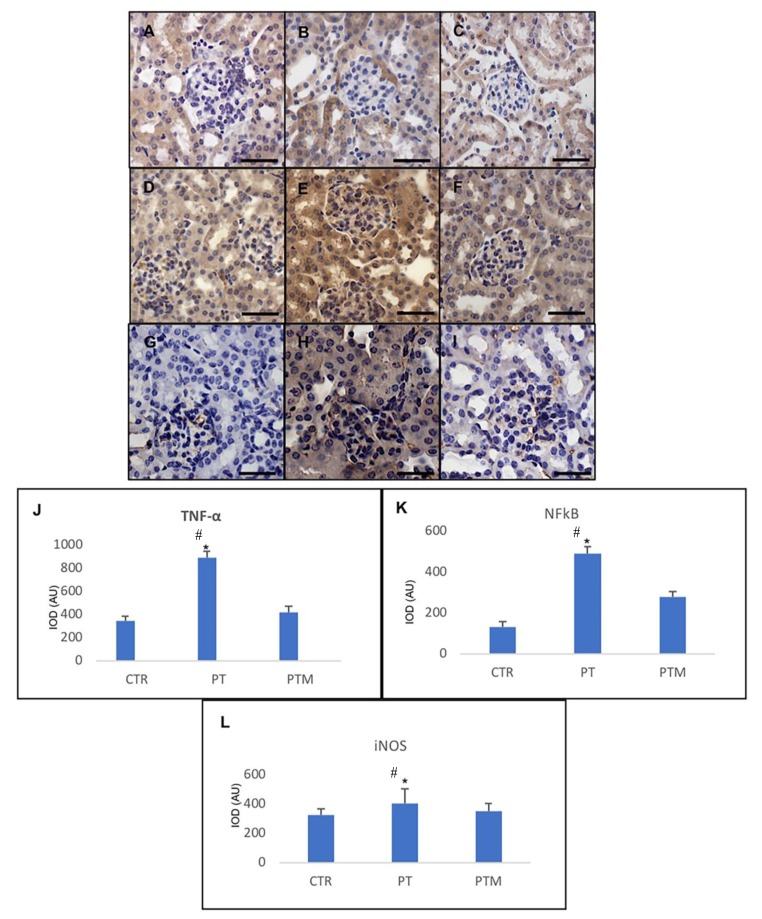
Photomicrographs showing transforming TNF-α (**A**–**C**), NF-kB (**D**–**F**) and iNOS (**G**–**I**) immunohistochemistry of control mice (**A**,**D**,**G**), pristane-LN mice (**B**,**E**,**H**) and pristane-LN mice treated with melatonin (**C**,**F**,**I**). Bar equals 20 μm. The graphs (**J**–**L**) summarizes proteins immunohistomorphometrical analysis of all experimental groups. * # indicate respectively statistically significant differences vs. control and pristane-LN mice treated with melatonin: *p* < 0.05. CTR: control mice PT: pristane-LN mice PTM: pristane-LN mice treated with melatonin.

An increased inflammatory and oxidative status has been detected in pristane-LN mice. In particular, kidney expression of tumor necrosis factor alpha (TNF-α), a crucial pro-inflammatory cytokine, was determined for brief evaluation of kidney inflammatory status caused by LN. The TNF- α levels were moderate/strong in pristane-LN mice in comparison with low levels of this protein in the kidney of control mice (Figure 5A,B). On the contrary to pristane-LN mice, significant TNF-α reduction was observed in the melatonin-treated LN group showing lower levels (Figure 5C).

Moreover, as shown in Figure 5D,E, renal proinflammatory NF-κB production was increased markedly in pristane-induced LN mice compared with control mice, while melatonin treatment significantly lowered the expression of NF-κB in LN mice kidneys (Figure 5F).

Immunohistochemical analysis of the critical inducible enzyme considered a marker of oxidative stress, inducible nitric oxide synthase (iNOS), which showed a significant increase of expression in kidneys of pristane-LN mice with respect to kidneys of control mice showing weak expression. Furthermore, kidneys of the pristane-LN mice treated with melatonin showed a significant reduction of this oxidative stress-related protein expression (Figure 5G–I).

All the observations reported above were confirmed by the histomorphometrical analysis of renal TNF-α (Figure 5J) NF-κB (Figure 5K) and iNOS (Figure 5L).

### 2.4. Kidney NLRP3 Evaluation

**Figure 6:**
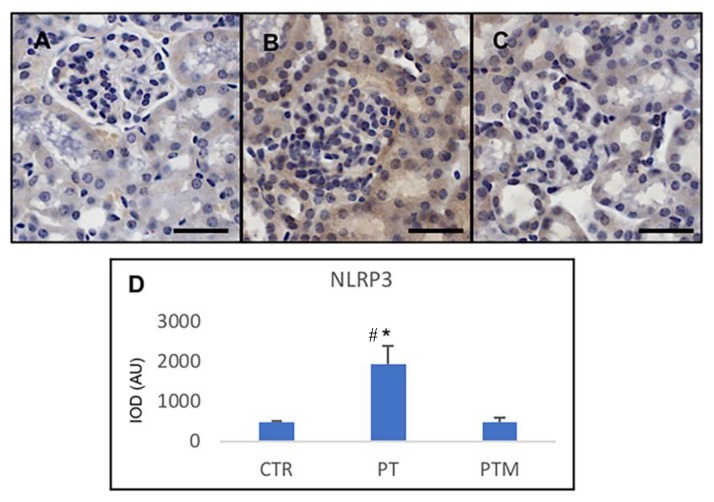
Photomicrographs showing transforming NLRP3 immunohistochemistry of control mice (**A**), pristane-LN mice (**B**) and pristane-LN mice treated with melatonin (**C**). Bar equals 20 μm. The graph (**D**) summarizes NLRP3 immunohistomorphometrical analysis of all experimental groups. * # indicate respectively statistically significant differences vs. control and pristane-LN mice treated with melatonin: * *p* < 0.05. CTR: control mice PT: pristane-LN mice PTM: pristane-LN mice treated with melatonin.

To further investigate the inflammatory pathway, the activation of the NLRP3-associated inflammasome was determined. NLRP3 levels were significantly higher in the pristane-induced LN mice compared with the normal control mice (*p* <0.05) (Figure 6). Melatonin treatment of pristane-induced LN mice significantly reduced the expression of this protein. The result illustrated that the NLRP3-mediated inflammasome pathway was effectively inhibited by melatonin treatment in the kidney of LN mice.

### 2.5. Capillary Integrity Evaluation

**Figure 7:**
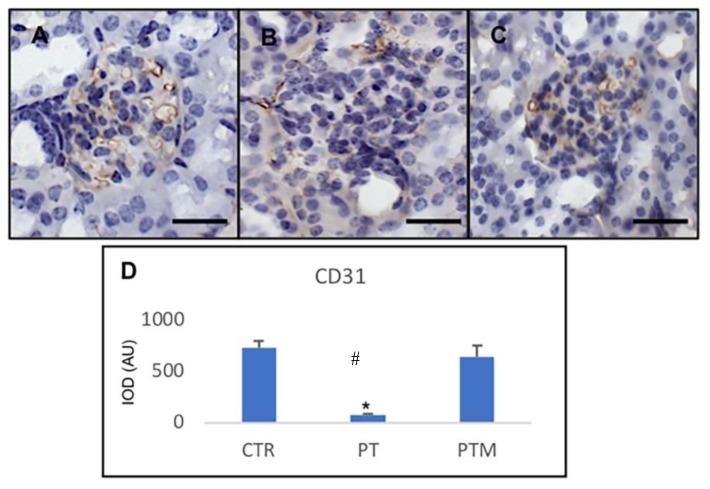
Photomicrographs showing transforming CD31 immunohistochemistry of control mice (**A**), pristane-LN mice (**B**) and pristane-LN mice treated with melatonin (**C**). Bar equals 20 μm. The graph (**D**) summarizes CD31 immunohistomorphometrical analysis of all experimental groups. * # indicate respectively statistically significant differences vs. control and pristane-LN mice treated with melatonin: * *p* < 0.05. CTR: control mice PT: pristane-LN mice PTM: pristane-LN mice treated with melatonin.

To measure vascular integrity, we identified capillaries via immunohistochemistry of a well-defined endothelial cell surface marker CD-31. CD-31 staining at the glomerular tuft level was confirmed in control mice kidneys, while low expression of this protein at the same level showed vascular rarefaction in pristane-induced LN mice. Treatment with melatonin restored vascular damage showing a positive intensity similar to control animals (Figure 7).

## 3. Discussion

In the present study, we investigated whether melatonin treatment could relieve the manifestation of lupus nephritis in the pristane-induced-LN mouse model together with its possible molecular mechanisms. Our previous findings showed that treatment with melatonin attenuated the deleterious effects of LN on renal function via the inhibition of inflammation and oxidative stress [26]. Our present findings showed a decreased expression of SIRT1 in pristane-induced-LN mice. This protein is an NAD-dependent protein deacetylase which participates on maintenance of proper renal physiology [27] due to its role in alleviation of a multitude of the NF-κB pathway-driven inflammation and metabolic disorders [28]. It negatively regulates inflammation interacting with NF-κB, the factor which regulates the transcription of pro-inflammatory cytokine genes, such as those coding for TNF-α and inflammatory interleukins. Indeed, in pristane-induced-LN mice, we detected increased tissue levels of TNF-α and glomerular damage accompanied by a reduction in endothelial CD31 and activated NF-κB/NLRP3 inflammasome signaling indicating an inflammatory response and tissue injury. These findings are in accordance with detected NF-κB activation in LN in humans [29] and animals [30]. The increased expression of NLRP3 stressed our previous results reporting the inflammatory status which led to apoptosis in the same animal model [26]. These morphological inflammatory changes affect almost all structures of the nephron. Similar alterations have been observed in patients with diabetic nephropathy, even before alterations in kidney filtration [31,32]. In particular, in these patients, changes in renal interstitium and tubules preceded glomerular damage and were associated with alteration in widely available biomarkers of kidney diseases such as NGAL, serum cystatine C and urine IgG [32]. Taken together, these findings emphasize the importance of activation of the NF-κB/NLRP3 inflammasome signaling as a key mechanism in LN disease. The NLRP3 inflammasome is a multiprotein oligomer activated by NF-κB which in turn promotes the maturation of interleukins, such as IL-1β and IL-18 [33]. Consistently, it was showed that serum cytokine levels were also elevated upon pristane induction of LN, which collectively supported the activation of the NLRP3 inflammasome in the kidney [34]. Moreover, increased ROS production could be considered as the main culprit behind the activation of the NF-κB/NLRP3 inflammasome signaling promoting the expression of iNOS [35] and, in this way, contributes to the progression of inflammation and oxidative stress. Accordingly, the antioxidant enzymes, superoxide dismutase (SOD) and catalase (CAT), were reduced in pristane-induced LN mice [26].

Immunosuppressants and corticosteroids have been considered the conventional therapy drug for SLE and lupus nephritis since the 1950s [36]. These molecules indeed act by pro-inflammatory cytokine inhibition, such as IL-1, IL-6 and TNF-α [37]. However, given the known dose-related and long-term-use adverse effects of these drugs and because patients are often refractory to these conventional treatments, we actually need to develop new alternative/adjunctive therapies for the treatment of LN with minimal doses or no corticosteroids. To date, therapy for LN has been evolving with a substantial number of trials of promising new agents based on the fundamental pathophysiological mechanisms which are centered on inflammation [17,26]. These compounds have to be efficacious, without side effects and maximize patient adherence. Among these potential molecules, we evaluated melatonin, a potent anti-inflammatory natural compound without important side effects [20,21,38,39].

Our results showed that melatonin preserves kidney morphology in this animal model at the glomerular level with decrease inflammation and preservation of endothelial CD31 expression and at the tubular level. In particular, this indolamine was able to prevent LN-induced activation of NF-κB/NLRP3 inflammasome signaling and, in this manner, ameliorate the inflammatory status of kidneys. It up-regulated SIRT1 [40,41] which in turn suppressed NF-κB signaling. As expected, we detected NLRP3 inflammasome downregulation resulting in the reduction of glomerular inflammation and proteinuria. Furthermore, Kawai et al. [42] demonstrated that SIRT1-mediated deacetylation of Nrf2, the factor involved in the regulation of antioxidant proteins, can antagonize oxidative and inflammatory damage in vitro. However, other studies have demonstrated that SIRT1 overexpression significantly promoted the nuclear accumulation, DNA binding and transcriptional activity of Nrf2 and Nrf2-mediated gene expression [43]. In vivo experiments confirmed the role of SIRT1-mediated Nrf2 regulation, improving antioxidant status in kidney [41]. So, to further explore the underlying mechanism, we determined the effect of melatonin on Nrf2 signaling in the kidneys of LN-induced mice. Our findings showed decreased expression of Nrf2 in LN, the effect that was reversed in melatonin-treated mice, confirming our previous results that showed a reduction of the endogenous antioxidant enzymes SOD and CAT [26]. For this reason, it is important to consider that melatonin-mediated SIRT1 activation suppresses inflammation through NF-κB/NLRP3 inflammasome signaling. Whatsmore, Nrf2 activation, mediated by melatonin activation of SIRT1, results in the inhibition of ROS and, therefore, it contributes to the inhibition of NF-κB/NLRP3 inflammasome signaling, too. In this way, attenuation of pro-inflammatory mediators and apoptosis occurs in the pristane-induced LN mice kidney. This is in accordance with previous studies, in which knock-down of Nrf2 led to NLRP3 activation in cerebral ischemia-reperfusion injury [44]. Furthermore, recently, Peng et al. [45] demonstrated that melatonin inhibited NLRP3 inflammasome activation via the SIRT1-Nrf2 pathway in the mouse model of chronic obstructive pulmonary disease. As the activation of NLRP3 inflammasome is promoted by NF-κB [33], the inhibition of NF-κB by Nrf2 can result in the suppression of NLRP3 inflammasome assembly [44].

**Figure 8:**
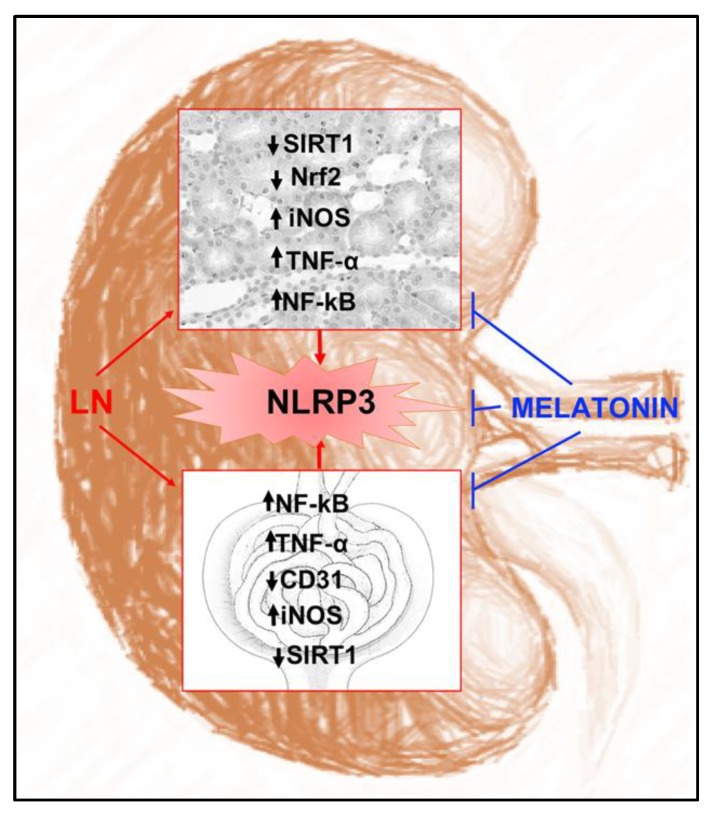
Melatonin protective effects against LN. Schematic representation of melatonin attenuation of kidney oxidative stress, inflammation and morphological alterations in pristane-LN mice.

In summary, our results demonstrate NF-κB/NLRP3 inflammasome activation with further inflammatory cytokine involvement, together with an increase in ROS in LN nephrotoxicity. Melatonin considerably prevented LN pathological events, particularly by attenuation of oxidative stress and inflammation. Its activation of SIRT1 induced Nrf2 antioxidant protection and inhibited ROS-induced activation of NF-κB/NLRP3 inflammasome signaling in the kidney of LN mice (Figure 8).

Therefore, our study confers new information on the protective mechanism of melatonin against LES nephrotoxicity.

Moreover, recently, it has been shown that the liver is an important target organ in SLE pathology. In this organ, during SLE, there is morphological damage characterized not only by hepatic IgG deposits, but also by a pro-inflammatory cytokine increase [46,47]. So, the results of this study could create the basis for future studies aimed to evaluate the potential anti-inflammatory effect of melatonin in the liver of the pristane-induced lupus model.

## 4. Materials and Methods

### 4.1. Animal Model

Fifty female mice BALB/c, at 8 weeks of age, were obtained from the Federal University of Pelotas (Pelotas, Brazil). The care of animals used in the present study followed local and international guidelines in accordance also with the recommendations by the Research and Ethics Committee on Health Research Group and Graduate Studies of Clinical Hospital of Porto Alegre (Brazil) (Ethical approvalnumber: 14-0588; date: 13th May 2015).

BALB/c mice were randomly divided into the following groups: (1) Pristane-induced LN mice (*n* = 10). Mice were intraperitoneally injected 500 μL of pristane (Sigma Aldrich, MO, USA) [26,45,48,49,50]; (2) Control mice (pristane vehicle) (*n* = 10). Mice were intraperitoneally injected 500 μL of saline; (3) Melatonin vehicle mice (*n* = 10). Mice were treated 6 months with 1% ethanol in drinking water; (4) Melatonin control mice (*n* = 10). Mice were treated 6 months with melatonin 10 mg/kg/day dissolved in 1% ethanol and added to drinking water; (5) Pristane-induced LN mice (*n* = 10). Mice were intraperitoneally injected 500 μL of pristane and they were, subsequently, treated for 6 months with melatonin 10 mg/kg/day dissolved in 1% ethanol and added to drinking water, starting 1 day after pristane induction of LN. Mice were sacrificed and the kidneys were acquired and processed for further analysis. For morphological and immunohistochemical analysis, the kidneys were dehydrated in ethanol and embedded in paraffin using standard protocol. Subsequently, the samples were sectioned to 7 μm using microtome.

### 4.2. Kidney Injury Score

Kidney paraffin-embedded sections were stained using haematoxiylin-eosin following the standard protocol. Subsequently, two independent, blinded to treatment researchers observed the kidneys with the optical microscopy (Olympus, Hamburg, Germany) at a final magnification of 400×. Kidney-embedded paraffin sections were stained with haematoxylin–eosin, according to standard protocol and then were observed with an optical microscopy (Olympus, Hamburg, Germany) at a final magnification of 400×. Kidney injury scores were assessed analyzing glomerular and tubular morphology, inflammatory cells infiltration, glomerular hypercellularity, proximal tubules brush border detachment, tubular lumen narrowing and epithelial tubules alterations. Further details about kidney injury scores are reported by Dos Santos et al. [26].

### 4.3. Periodic Acid SCHIFF (PAS) Staining

Kidney paraffin-embedded sections were stained using PAS staining and intraglomerular mesangial proliferation together with proximal tubule brush boarder alterations were analyzed [51,52]. A minimum of 50 renal fields per animal were observed using light microscope (Olympus, Germany) at final magnification of 400×. The pink color was measured using Image Pro software (Image Pro Premier 9.1, Media Cybernetics Inc., Rockville USA), separately at glomerular and tubular level. All analysis were conducted in a blind fashion.

### 4.4. Morphometry

The sections stained with periodic acid-Schiff (PAS; Schiff’s reagent; Sigma-Aldrich) were used for measurement of mean glomerular tuft volume (GV) and glomerular cross-sectional area (GA). GV was determined from (GA) by light microscopy as described previously. Photomicrographs were scanned, and profile areas were traced using an image analyzer (Image-Pro Plus™, Media Cybernetics, Silver Spring, MD). GA was determined as the average area of a total of 30 cortical glomeruli. GV was calculated as

GV = β/k × (GA) 3/2(1)

where β = 1.38, which is the shape coefficient for spheres (the idealized shape of glomeruli), and k = 1.1, which is a size distribution coefficient. To quantify mesangial expansion, 20 glomeruli cut at their vascular pole were used in each animal. Mesangial (MMA) area was defined as PAS-positive and nuclei-free area in mesangium [53,54].

### 4.5. Immunohistochemical Analysis

For immunohistochemical analysis, sections of kidneys were processed according to an avidin complexed with biotinylated peroxidase technique (ABC/HRP). The sections, after dehydration, were immersed in citrate buffer (pH 6) and subjected to microwave irradiation twice for 5 min. Subsequently, for quenching endogenous peroxidase activity, all sections were immersed for 30 min in 0.3% hydrogen peroxide in methanol. To block non-specific binding, the slides were incubated for 1 h at room temperature with mouse Ig-blocking reagent (Vector Laboratories, Burlingame, CA, USA). The sections were then incubated 1 h at 37 °C with primary antibodies at concentrations assessed in preliminary experiments, in particular rabbit anti-SIRT1 polyclonal antibody (1:100, Santa Cruz Biotechnology, Santa Cruz, CA, USA); rabbit anti-Nrf2 polyclonal antibody (1:100, Abcam); goat anti-TNF-α polyclonal antibody (1:200, Santa Cruz Biotechnology); rabbit anti-NF-kB polyclonal antibody (1:100, Abcam); rabbit anti-iNOS polyclonal antibody (1:100, Santa Cruz Biotechnology); rabbit anti-NLRP3 polyclonal antibody (1:500, Abcam) and rat anti-CD-31 antibody (1:100, Serotec). Samples, after incubation with primary antibodies, were rinsed twice in PBS, exposed for 1 h at room temperature to the appropriate secondary biotinylated goat anti-mouse or anti-rabbit IgG (Vector Laboratories, BA9200 and BA1000) and then treated with peroxidase-conjugated avidin (Vectastain Elite ABC Kit Standard* PK 6-100) for 35 min. After this step, the slides were treated with 0.05% 3,3-diaminobenzidine (DAB) with 0.1% H2O2 (DAB substrate kit for peroxidase, Vector Laboratories SK-4100). The sections were counterstained with Mayer’s haematoxylin and observed using a light microscope. Negative control experiments were carried out without the primary antibody incubation. The staining was evaluated by two competent observers, and the immunopositivity was assessed microdensitometrically using an image analyzer (Image-Pro Plus™, Media Cybernetics, Silver Spring, MD, USA) connected to the microscope by a camera. The software has a function that allows quantifying the integrated optical density (IOD) measurements. Briefly, after the grey-scale conversion of the images, we set the threshold values of black and white and created the optical density-scale calibration using known algorithms. This calibration was then applied for each capture imported in the software for processing. The background obtained in sections exposed to non-immune serum was considered as zero in system calibration. In each section, ten areas were considered. Statistical significance of differences between the experimental groups was estimated using ANOVA and Bonferroni tests with *p* < 0.05 considered significant.

### 4.6. Statistical Analysis

The data are expressed as mean ± standard error of the mean (SEM). Data for multiple variable comparisons were analyzed by one-way analysis of variance (ANOVA corrected Bonferroni test). *p* ≤ 0.05 is considered significant for all statistical analysis performed in this study.

## Abbreviations

SLE	Systemic lupus erythematosus
LN	Lupus nephritis
NLRP3	NOD-like receptor protein 3
NF-kB	Nuclear factor kappa B
iNOS	Inducible nitric oxide synthase
SIRT1	Sirtuin 1
SOD	Superoxide dismutase
CAT	Catalase
TNF-α	Tumor necrosis factor alpha

## References

[1] Hanly J.G., O’Keeffe A.G., Su L., Urowitz M.B., Romero-Diaz J., Gordon C., Bae S.C., Bernatsky S., Clarke A.E., Wallace D.J. (2016). The frequency and outcome of lupus nephritis: Results from an international inception cohort study. Rheumatology.

[2] Mistry P., Kaplan M.J. (2017). Cell death in the pathogenesis of systemic lupus erythematosus and lupus nephritis. Clin. Immunol..

[3] Dedong H., Feiyan Z., Jie S., Xiaowei L., Shaoyang W. (2019). Analysis of interleukin-17 and interleukin-23 for estimating disease activity and predicting the response to treatment in active lupus nephritis patients. Immunol. Lett..

[4] Li D., Shi G., Wang J., Zhang D., Pan Y., Dou H., Hou Y. (2019). Baicalein ameliorates pristane-induced lupus nephritis via activating Nrf2/HO-1 in myeloid-derived suppressor cells. Arthritis Res. Ther..

[5] Batten M., Ramamoorthi N., Kljavin N.M., Ma C.S., Cox J.H., Dengler H.S., Danilenko D.M., Caplazi P., Wong M., Fulcher D.A. (2010). IL-27 supports germinal center function by enhancing IL-21 production and the function of T follicular helper cells. J. Exp. Med..

[6] Satoh M., Reeves W.H. (1994). Induction of lupus-associated autoantibodies in BALB/c mice by intraperitoneal injection of pristane. J. Exp. Med..

[7] Gluhovschi C., Gluhovschi G., Potencz E., Herman D., Trandafirescu V., Petrica L., Velciov S., Bozdog G., Bob F., Vernic C. (2010). The endothelial cell markers von Willebrand Factor (vWF), CD31 and CD34 are lost in glomerulonephritis and no longer correlate with the morphological indices of glomerular sclerosis, interstitial fibrosis, activity and chronicity. Folia Histochem. Cytobiol..

[8] El-Banawy H.S., Gaber E.W., Maharem D.A., Matrawy K.A. (2012). Angiopoietin-2, endothelial dysfunction and renal involvement in patients with systemic lupus erythematosus. J. Nephrol..

[9] Pawar R.D., Goilav B., Xia Y., Zhuang H., Herlitz L., Reeves W.H., Putterman C. (2014). Serum autoantibodies in pristane-induced lupus are regulated by neutrophil gelatinase associated lipocalin. Clin. Immunol..

[10] Sporek M., Dumnicka P., Gala-Bladzinska A., Ceranowicz P., Warzecha Z., Dembinski A., Stepien E., Walocha J., Drozdz R., Kuzniewski M. (2016). Angiopoietin-2 Is an Early Indicator of Acute Pancreatic-Renal Syndrome in Patients with Acute Pancreatitis. Mediat. Inflamm..

[11] Ding Y., Nie L.M., Pang Y., Wu W.J., Tan Y., Yu F., Zhao M.H. (2018). Composite urinary biomarkers to predict pathological tubulointerstitial lesions in lupus nephritis. Lupus.

[12] Kahlenberg J.M., Thacker S.G., Berthier C.C., Cohen C.D., Kretzler M., Kaplan M.J. (2011). Inflammasome activation of IL-18 results in endothelial progenitor cell dysfunction in systemic lupus erythematosus. J. Immunol..

[13] Fu R., Guo C., Wang S., Huang Y., Jin O., Hu H., Chen J., Xu B., Zhou M., Zhao J. (2017). Podocyte Activation of NLRP3 Inflammasomes Contributes to the Development of Proteinuria in Lupus Nephritis. Arthritis Rheumatol..

[14] Ma Z.Z., Sun H.S., Lv J.C., Guo L., Yang Q.R. (2018). Expression and clinical significance of the NEK7-NLRP3 inflammasome signaling pathway in patients with systemic lupus erythematosus. J. Inflamm..

[15] Kahlenberg J.M., Carmona-Rivera C., Smith C.K., Kaplan M.J. (2013). Neutrophil extracellular trap-associated protein activation of the NLRP3 inflammasome is enhanced in lupus macrophages. J. Immunol..

[16] Su B., Ye H., You X., Ni H., Chen X., Li L. (2018). Icariin alleviates murine lupus nephritis via inhibiting NF-κB activation pathway and NLRP3 inflammasome. Life Sci..

[17] Zhao J., Wang J., Zhou M., Li M., Li M., Tan H. (2019). Curcumin attenuates murine lupus via inhibiting NLRP3 inflammasome. Int. Immunopharmacol..

[18] Rahim I., Djerdjouri B., Sayed R.K., Fernández-Ortiz M., Fernández-Gil B., Hidalgo-Gutiérrez A., López L.C., Escames G., Reiter R.J., Acuña-Castroviejo D. (2017). Melatonin administration to wild-type mice and nontreated NLRP3 mutant mice share similar inhibition of the inflammatory response during sepsis. J. Pineal Res..

[19] Galano A., Reiter R.J. (2018). Melatonin and its metabolites vs. oxidative stress: From individual actions to collective protection. J. Pineal Res..

[20] Favero G., Franceschetti L., Bonomini F., Rodella L.F., Rezzani R. (2017). Melatonin as an Anti-Inflammatory Agent Modulating Inflammasome Activation. Int. J. Endocrinol..

[21] Jaworek J., Leja-Szpak A., Nawrot-Porąbka K., Szklarczyk J., Kot M., Pierzchalski P., Góralska M., Ceranowicz P., Warzecha Z., Dembinski A. (2017). Effects of Melatonin and Its Analogues on Pancreatic Inflammation, Enzyme Secretion, and Tumorigenesis. Int. J. Mol. Sci..

[22] Favero G., Trapletti V., Bonomini F., Stacchiotti A., Lavazza A., Rodella L.F., Rezzani R. (2017). Oral Supplementation of Melatonin Protects against Fibromyalgia-Related Skeletal Muscle Alterations in Reserpine-Induced Myalgia Rats. Int. J. Mol. Sci..

[23] Dos Santos M., Poletti P.T., Favero G., Stacchiotti A., Bonomini F., Montanari C.C., Bona S.R., Marroni N.P., Rezzani R., Veronese F.V. (2018). Protective effects of quercetin treatment in a pristane-induced mouse model of lupus nephritis. Autoimmunity.

[24] Moniruzzaman M., Ghosal I., Das D., Chakraborty S.B. (2018). Melatonin ameliorates H_2_O_2_-induced oxidative stress through modulation of Erk/Akt/NFkB pathway. Biol. Res..

[25] Hardeland R. (2019). Aging, Melatonin, and the Pro- and Anti-Inflammatory Networks. Int. J. Mol. Sci..

[26] Dos Santos M., Favero G., Bonomini F., Stacchiotti A., Rodella L.F., Veronese F.V., Rezzani R. (2018). Oral supplementation of melatonin protects against lupus nephritis renal injury in a pristane-induced lupus mouse model. Life Sci..

[27] Gao R., Chen J., Hu Y., Li Z., Wang S., Shetty S., Fu J. (2014). Sirt1 deletion leads to enhanced inflammation and aggravates endotoxin-induced acute kidney injury. PLoS ONE.

[28] Kauppinen A., Suuronen T., Ojala J., Kaarniranta K., Salminen A. (2013). Antagonistic crosstalk between NF-κB and SIRT1 in the regulation of inflammation and metabolic disorders. Cell. Signal..

[29] Zheng C.Z., Shu Y.B., Luo Y.L., Luo J. (2017). The role of miR-146a in modulating TRAF6-induced inflammation during lupus nephritis. Eur. Rev. Med. Pharmacol. Sci..

[30] Chalmers S.A., Garcia S.J., Reynolds J.A., Herlitz L., Putterman C. (2019). NF-kB signaling in myeloid cells mediates the pathogenesis of immune-mediated nephritis. J. Autoimmun..

[31] Ilyas Z., Chaiban J.T., Krikorian A. (2017). Novel insights into the pathophysiology and clinical aspects of diabetic nephropathy. Rev. Endocr. Metab. Disord..

[32] Żyłka A., Dumnicka P., Kuśnierz-Cabala B., Gala-Błądzińska A., Ceranowicz P., Kucharz J., Ząbek-Adamska A., Maziarz B., Drożdż R., Kuźniewski M. (2018). Markers of Glomerular and Tubular Damage in the Early Stage of Kidney Disease in Type 2 Diabetic Patients. Mediat. Inflamm..

[33] Zhang H., Liu L., Li L. (2018). Lentivirus-mediated knockdown of FcγRI (CD64) attenuated lupus nephritis via inhibition of NF-κB regulating NLRP3 inflammasome activation in MRL/lpr mice. J. Pharmacol. Sci..

[34] Qu H., Bian W., Xu Y. (2014). A novel NF-κB inhibitor, DHMEQ, ameliorates pristane-induced lupus in mice. Exp. Ther. Med..

[35] Morgan M.J., Liu Z.G. (2011). Crosstalk of reactive oxygen species and NF-κB signaling. Cell Res..

[36] Jacobson W.E., Heller B.I., Hammarsten J.F. (1951). The effect of cortisone in glomerulonephritis and the nephropathy of disseminated lupus erythematosus. Minn. Med..

[37] Lightstone L. (2013). Minimising steroids in lupus nephritis--will B cell depletion pave the way?. Lupus.

[38] Ostjen C.A., Rosa C.G.S., Hartmann R.M., Schemitt E.G., Colares J.R., Marroni N.P. (2019). Anti-inflammatory and antioxidant effect of melatonin on recovery from muscular trauma induced in rats. Exp. Mol. Pathol..

[39] Andersen L.P., Gögenur I., Rosenberg J., Reiter R.J. (2016). The Safety of Melatonin in Humans. Clin. Drug Investig..

[40] Shah S.A., Khan M., Jo M.H., Jo M.G., Amin F.U., Kim M.O. (2017). Melatonin Stimulates the SIRT1/Nrf2 Signaling Pathway Counteracting Lipopolysaccharide (LPS)-Induced Oxidative Stress to Rescue Postnatal Rat Brain. CNS Neurosci. Ther..

[41] Shi S., Lei S., Tang C., Wang K., Xia Z. (2019). Melatonin attenuates acute kidney ischemia/reperfusion injury in diabetic rats by activation of the SIRT1/Nrf2/HO-1 signaling pathway. Biosci. Rep..

[42] Kawai Y., Garduño L., Theodore M., Yang J., Arinze I.J. (2011). Acetylation-deacetylation of the transcription factor Nrf2 (nuclear factor erythroid 2-related factor 2) regulates its transcriptional activity and nucleocytoplasmic localization. J. Biol. Chem..

[43] Li S., Zhao G., Chen L., Ding Y., Lian J., Hong G., Lu Z. (2016). Resveratrol protects mice from paraquat-induced lung injury: The important role of SIRT1 and NRF2 antioxidant pathways. Mol. Med. Rep..

[44] Hou Y., Wang Y., He Q., Li L., Xie H., Zhao Y., Zhao J. (2018). Nrf2 inhibits NLRP3 inflammasome activation through regulating Trx1/TXNIP complex in cerebral ischemia reperfusion injury. Behav. Brain Res..

[45] Peng Z., Zhang W., Qiao J., He B. (2018). Melatonin attenuates airway inflammation via SIRT1 dependent inhibition of NLRP3 inflammasome and IL-1β in rats with COPD. Int. Immunopharmacol..

[46] Adiga A., Nugent K. (2017). Lupus Hepatitis and Autoimmune Hepatitis (Lupoid Hepatitis). Am. J. Med. Sci..

[47] Fang X., Zaman M.H., Guo X., Ding H., Xie C., Zhang X., Deng G.M. (2018). Role of Hepatic Deposited Immunoglobulin G in the Pathogenesis of Liver Damage in Systemic Lupus Erythematosus. Front. Immunol..

[48] Satoh M., Kumar A., Kanwar Y.S., Reeves W.H. (1995). Anti-nuclear antibody production and immune-complex glomerulonephritis in BALB/c mice treated with pristane. Proc. Natl. Acad. Sci. USA.

[49] Liu C., Kanamaru Y., Watanabe T., Tada N., Horikoshi S., Suzuki Y., Liu Z., Tomino Y. (2015). Targeted IgA Fc receptor I (FcαRI) therapy in the early intervention and treatment of pristane-induced lupus nephritis in mice. Clin. Exp. Immunol..

[50] Lin Y., Yan Y., Zhang H., Chen Y., He Y., Wang S., Fang L., Lv Y., Du G. (2017). Salvianolic acid A alleviates renal injury in systemic lupus erythematosus induced by pristane in BALB/c mice. Acta Pharm. Sin. B.

[51] Stacchiotti A., Favero G., Giugno L., Lavazza A., Reiter R.J., Rodella L.F., Rezzani R. (2014). Mitochondrial and metabolic dysfunction in renal convoluted tubules of obese mice: Protective role of melatonin. PLoS ONE.

[52] Nakagawa P., Masjoan-Juncos J.X., Basha H., Janic B., Worou M.E., Liao T.D., Romero C.A., Peterson E.L., Carretero O.A. (2017). Effects of N-acetyl-seryl-asparyl-lysyl-proline on blood pressure, renal damage, and mortality in systemic lupus erythematosus. Physiol. Rep..

[53] Samnegård B., Jacobson S.H., Jaremko G., Johansson B.L., Ekberg K., Isaksson B., Eriksson L., Wahren J., Sjöquist M. (2005). C-peptide prevents glomerular hypertrophy and mesangial matrix expansion in diabetic rats. Nephrol. Dial. Transplant..

[54] Malatiali S., Francis I., Barac-Nieto M. (2017). Insulin Prevents Hyperfiltration and Proteinuria but Not Glomerular Hypertrophy and Increases Mesangial Matrix Expansion in Diabetic Rats. Med. Princ. Pract..

